# Laparoscopic Sentinel Lymph Node Biopsy for Prostate Cancer: The Relevance of Locations Outside the Extended Dissection Area

**DOI:** 10.1155/2012/751753

**Published:** 2011-09-19

**Authors:** W. Meinhardt, H. G. van der Poel, R. A. Valdés Olmos, A. Bex, O. R. Brouwer, S. Horenblas

**Affiliations:** ^1^Department of Urology, Netherlands Cancer Institute/Antoni van Leeuwenhoek Hospital, Plesmanlaan 121, 1066 CX Amsterdam, The Netherlands; ^2^Department of Nuclear Medicine, Netherlands Cancer Institute/Antoni van Leeuwenhoek Hospital, Plesmanlaan 121, 1066 CX Amsterdam, The Netherlands

## Abstract

*Objective*. To assess the relevance of sentinel lymph nodes (SNs) outside the extended pelvic lymph node dissection area (e-PLND). *Patients and Methods*. Evaluation of our laparoscopic SN procedures for prostate cancer patients of intermediate prognosis. Retrospective data collection on the exact location of the excised SNs and the pathology results were analyzed. *Results and Limitations*. Of the 121 patients, 49 had positive lymph nodes. 37 patients (31%) had SNs outside the e-PLND template. Five of these nodes were tumor bearing but only twice exclusively so. Of the 14 patients considered for salvage treatment, 6 were node positive. 7 of these 14 patients (50%) had SNs outside the extended dissection area, yet none of these nodes were tumor positive. Limitations are those of a retrospective study. *Conclusions*. Laparoscopic SN biopsy may show SNs outside the e-PLND template in 31% of the patients. However, nodes that are exclusively positive in one of these areas are rare. For the dichotomy positive or negative nodes, the locations outside the e-PLND area are not often relevant. Nevertheless, when all positive nodes are to be treated by resection or radiotherapy, these locations are relevant. When considering salvage treatment for prostate cancer, the method is feasible.

## 1. Introduction

Sentinel node (SN) biopsy for prostate cancer has been validated in open surgery, combined with a prostatectomy, as well as in laparoscopic surgery [1–3].

The SN concept is based on the concept of sequential metastatic spread, starting with 1 or more nodes on a direct drainage pathway from the primary tumor site. A negative tumor status of the SN is equivalent to the absence of lymphatic involvement. The SN method does not pretend to identify all tumor-bearing nodes. 

Distinguishing the SN from second echelon nodes on only one preoperative image can be difficult in the pelvic region, since lymphatic channels are seldom visualized. An alternative way to make the distinction between the SN and second echelon nodes is by acquiring several sequential images and use the order of appearance as a criterion. 

Compared to an extended pelvic lymph node dissection (e-PLND), SN biopsy has the advantage that it enables identification of SNs outside the e-PLND area [3, 4]. 

In the present study, our experience with this aspect is presented.

## 2. Patients and Methods

### 2.1. Patients

According to the EAU guidelines, we perform a laparoscopic SN procedure only in prostate cancer patients for whom the results will influence treatment decisions [5]. Patients who opt for external beam radiation therapy are candidates for a laparoscopic SN biopsy, when their PSA is >10, Gleason >6 or Stage >T2b. Depending on the nodal status, we adjust the radiation target volume and the duration of the hormonal therapy. Other candidates are patients who had local treatment failure and consider salvage treatment of the prostate, since positive nodes make us refrain from salvage prostate treatment.

### 2.2. Preoperative Imaging

Fifteen minutes after transrectal injection of 99mTechnetium-nanocolloid (Amersham Cygne, Eindhoven, The Netherlands) into both lobes of the prostate, guided by ultrasound, the first static planar lymphoscintigraphic image is acquired. This is repeated after 2 hours, and by comparing the 2 images, the distinction can be made between SNs and second-echelon nodes. The first lymph nodes in each station appearing on early planar imaging were considered to be the SNs. Nodes appearing later in the same stations or cranially to the previously identified SNs were considered to be second-echelon nodes. In addition, SPECT/CT (Symbia T, Siemens, Erlangen, Germany) was performed at 2 hours after injection. After image fusion, SNs were anatomically localized. If the SPECT/CT showed more lymph nodes compared to the planar images, the same criteria to distinguish first from second echelon were applied.

### 2.3. Surgical Procedure

**Figure 1:**
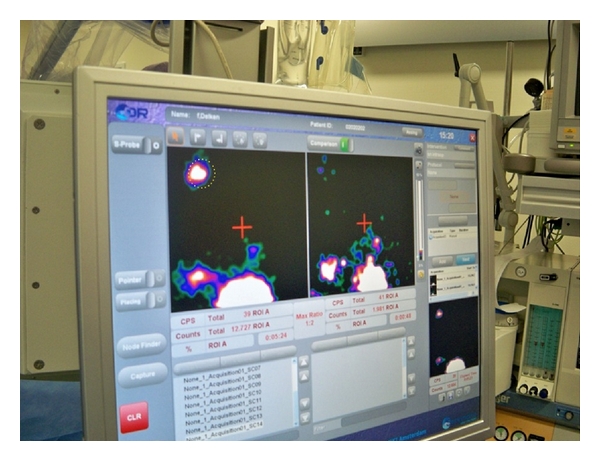
Combined intraoperative surgical guidance using the portable gamma camera and the laparoscopic gamma probe. On the screen, the iodine seed on the tip of the probe is represented by a circle, before (left) and after (right) excision of an SN.

A laparoscopic gamma probe (EuroProbe, Euro medical Instruments, London, UK) was used to acoustically localize radioactive nodes during the operation. After the first 40 patients we refined the method by the use of a portable gamma camera (Sentinella, Oncovision, Valencia, Spain) [6] (Figure 1). The portable gamma camera can visualize the radioactive hotspots on screen and can be used to guide the gamma probe in the direction of the SN by placing a radioactive iodine seed on the tip of the gamma probe, which can be depicted separately on screen (Figure 1). In addition, if the gamma camera showed residual focal radioactivity after the removal of an SN at the same location, it was considered as another possible SN and was also removed. *Ex vivo*, the removed tissue was also examined with the gamma probe to confirm the excised SN is radioactive and to separate the radioactive SNs from adjacent tissue. 

In case of unilateral nonvisualization of SNs at preoperative imaging, a lymph-node dissection comprising the region around the bifurcation of the common iliac artery, including nodes of the common iliac, the internal iliac, the external iliac, and the obturator regions was performed on that side. The rationale for this is the possibility of nonvisualization in case of major tumor involvement of a lymph node, leading to rerouting of the 99mTechnetium-nanocolloid containing lymphatic.

In the first 35 patients, a confirmatory laparoscopic e-PLND was performed in order to evaluate the reliability of our SN method. The areas resected were around the external iliac artery and vein, the common iliac up to the crossing of the ureter, the internal iliac just passed the superior vesical artery, and the obturator fossa. This series has been reported before and showed no false-negative results [2].

 After the first 35 patients, we abandoned the confirmatory e-PLND, since we considered our SN method to be reliable.

## 3. Results

Between December 2005 and October 2010, 121 patients were treated. Of these 121 patients, 49 (40%) were node positive. In 37 patients (31%), SNs were identified and excised outside the e-PLND template, at the following locations: presacral, Cloquet's node, inguinal, para-aortic, abdominal wall, pararectal, behind the common iliac artery, and lateral to the external iliac artery. In five patients, these nodes were tumor positive (10% of the node positive patients and 4% of all patients). However, in merely two cases, this node was the only positive node retrieved from those patients. SN results are listed in Table 1. 

In case of unilateral nonvisualization of SNs, a dissection on that side revealed positive lymph nodes in 4 of 12 cases.

Characteristics of patients considered for salvage treatment in case of a prostate cancer recurrence are shown in Table 2. 6 of the 14 patients were node positive. In 7 of these 14 patients, we retrieved nodes outside the extended dissection area; however, none of these nodes were tumor positive. So, although in salvage candidates the occurrence of SNs outside the extended template is higher (50% versus 28%), harvesting these nodes did not result in change of treatment.

## 4. Discussion

By comparing the early and late static planar images, we make the distinction between SNs and second-echelon nodes. If this distinction is not made, we agree with Weckermann that it is better to avoid the term SN biopsy but use “radio guided surgery” instead [7]. The SN technique is designed to individualize the diagnostics and the therapeutic decisions. Pooling these data to draw conclusions on what an extended dissection area might be, or which area should be radiated, results in unpractical large areas [8]. 

The original application of the SN concept in melanoma and penile cancer often shows 1 or 2 lymphatic vessels leading to SNs [9, 10]. In the pelvic region, this is seldomly the case. Results of a recent study in bladder carcinoma illustrate this. Only the healthy side of the bladder was injected with tracer and crossing to the contralateral side was often found even to contralateral tumor bearing nodes [11]. In the pelvis, it is more appropriate to consider the lymphatics as a reticulum with only a few valves. 

Blockage of the lymph flow by tumor in the node may lead to nonvisualization and rerouting and even retrograde lymph flow of the 99mTechnetium-nanocolloid containing lymphatic flow. This concept has been visualized with SPECT/CT [12]. In our patients, it is illustrated by the fact that in the case of nonvisualization on one particular side, positive nodes on that side were found in a third of those cases. 

The appearance of radioactive nodes in the abdominal wall near the umbilical ligament, of pararectal nodes, of direct drainage to Cloquet's nodes and even to inguinal nodes may be explained by the above-named mechanisms. Tumor-positive nodes of prostate cancer in the inguinal region have been reported before by others. This may also be explained by leakage of the tracer during the injection, transrectally near the linea dentata. However, inguinal nodes have been reported without any transrectal injections [13, 14]. This leakage may also explain the drainage to pararectal nodes, which is often reported but hazardous to dissect laparoscopically. 

Radioactive lymphnodes are regularly identified outside the extended resection area more cranially and around the aorta and the vena cava. This may be explained by the regular lymph flow, and often these nodes might well be second-echelon nodes. In older studies, the presacral area is already included in the regular drainage regions of the prostate [15]. However, laparoscopic excision of these nodes may also be challenging. 

Salvage treatment of the prostate may result in serious complications. Identifying even micrometastases is important, since the balance between the potential benefits and risks for this type of treatment is different. We only consider salvage prostate treatment when the prostate is the only tumor-bearing site. As lymph node micrometastases cannot be accurately visualized using current imaging modalities, removal of lymph nodes for microscopic investigation is still indicated [16]. The usual parameters to stratify patients in risk groups do not apply to patients with a recurrence in their prostate, as illustrated by our high percentage of positive nodes in this group.

## 5. Conclusions

In prostate carcinoma, the laparoscopic SN technique identified positive lymph nodes outside the e-PLND area in 31% of the cases. They were the only site of tumor bearing nodes in 4% of the patients with positive nodes. For diagnostic purposes only, this makes these locations less relevant. However, for dissection or radiation of lymph nodes with curative intent, these locations are relevant, since they contained tumor in 10% of node positive patients. When considering salvage treatment for prostate cancer, the method is feasible, and in almost half of these patients, the results have led to a change in therapeutic decisions.

## Figures and Tables

**Table 1:** Location and pathology results of SNs outside the extended resection area. No = 37 patients (of the 121).

Location	Number of patients	Tumor bearing	Was it the only positive node retrieved?
Presacral	9	1	Yes
Cloquet's node	8	1	No
Inguinal	7	0	
Para-aortic	6	1	No
Abdominal wall	4	1	No
Pararectal	1	0	
Behind the common iliac artery	1	0	
Lateral to the external iliac artery	1	1	Yes

**Table 2:** Characteristics of 14 patients with a recurrence in the prostate and the outcome of the SN procedure. None of the nodes outside the extended dissection area was tumor positive.

Age	Previous treatment and time passed	PSA at time of recurrence	Gleason score	Outcome +: tumor bearing nodes	Location outside of extended dissection area
53 y	External beam, 9 y	2.2	7	−	−
68 y	Brachy therapy, 8 y	17	7	−	Para-aortic
68 y	External beam, 5 y	4.0	6	−	−
70 y	External beam, 8 y	4.8	8	+	−
63 y	Brachy therapy, 6 y	2.1	6	−	−
65 y	Brachy therapy, 7 y	11	7	−	Abdominal wall, near umbilical ligament
73 y	Brachy therapy, 2 y	21	6	+	Inguinal node (negative)
64 y	External beam, 3 y	7	7	+	−
61 y	External beam, 5 y	12.7	8	+	Para-aortic (negative)
62 y	HIFU, 2 y	30	8	−	inguinal
69 y	External beam, 3 y	3.8	7	+	Para-aortic (negative)
63 y	HIFU, 1 y	5.4	6	−	Abd. Wall, next to umbilical ligament
71 y	External beam, 6 y	1.7	7	−	
61 y	Brachy therapy, 5 y	2.1	9	+	
